# Ultra-low foetal radiation exposure in ^18^F-FDG PET/CT imaging with a long axial field-of-view PET/CT system

**DOI:** 10.1186/s40658-024-00648-w

**Published:** 2024-05-24

**Authors:** Charlotte L. C. Smith, Maqsood Yaqub, Ruud H. H. Wellenberg, Jelijn J. Knip, Ronald Boellaard, Gerben J. C. Zwezerijnen

**Affiliations:** 1 Department of Radiology and Nuclear Medicine, Amsterdam UMC location Vrije Universiteit Amsterdam, Boelelaan, Amsterdam, 1117 The Netherlands; 2 Cancer Center Amsterdam, Imaging and Biomarkers, Amsterdam, The Netherlands; 3 Department of Medical Oncology, Amsterdam UMC location Vrije Universiteit Amsterdam, Boelelaan, Amsterdam, 1117 The Netherlands

**Keywords:** Foetal radiation dose, Ultra-low PET/CT, LAFOV PET/CT, ^18^F-FDG PET, Pregnancy

## Abstract

**Purpose:**

Long axial field-of-view (LAFOV) PET/CT systems enable PET/CT scans with reduced injected activities because of improved sensitivity. With this study, we aimed to examine the foetal radiation dose from an ^18^F-FDG PET/CT scan on a LAFOV PET/CT system with reduced injected activity.

**Methods:**

Two pregnant women were retrospectively included and received an ^18^F-FDG PET/CT scan on a LAFOV PET/CT system with an intravenous bolus injection of 0.30 MBq/kg. Foetal radiation exposure from the PET was estimated using dose conversion factors from three published papers. Radiation exposure from the CT scans was estimated using CT-Expo.

**Results:**

Foetal radiation dose from the PET scan ranged between 0.11 and 0.44 mGy. Foetal radiation exposure from the CT scan ranged between < 0.10 – 0.90 mGy depending if the foetus was included in the field-of-view.

**Conclusion:**

Foetal radiation dose could be reduced to < 1.5 mGy when scanning pregnant patients on a LAFOV PET/CT system. The radiation dose to the foetus was reduced significantly in our study due to the increased sensitivity of the LAFOV PET/CT system.

## Introduction

^18^F-fluoro-deoxy-glucose (^18^F-FDG) positron emission tomography-computed tomography (PET/CT) imaging is widely used in oncology for diagnoses, staging and treatment response evaluation [1, 2]. However, in pregnant patients, the use of ^18^F-FDG PET/CT imaging can cause a dilemma because of the radiation exposure to the foetus [3, 4]. Therefore, it is crucial to minimize the radiation exposure to the foetus when performing an ^18^F-FDG PET/CT scan. Fortunately, the new long axial field-of-view (LAFOV) PET/CT systems exhibit ultra-high sensitivity compared to conventional short axial field-of-view (SAFOV) PET/CT systems, enabling substantial reductions in radioactive tracer dosages [5]. Reducing the amount of injected ^18^F-FDG activity will subsequently result in a reduced foetal radiation burden.

With this study, we aimed to report the foetal radiation dose when performing an ^18^F-FDG PET/CT scan on a LAFOV PET/CT system with reduced injected activity.

## Materials and methods

**Fig. 1:**
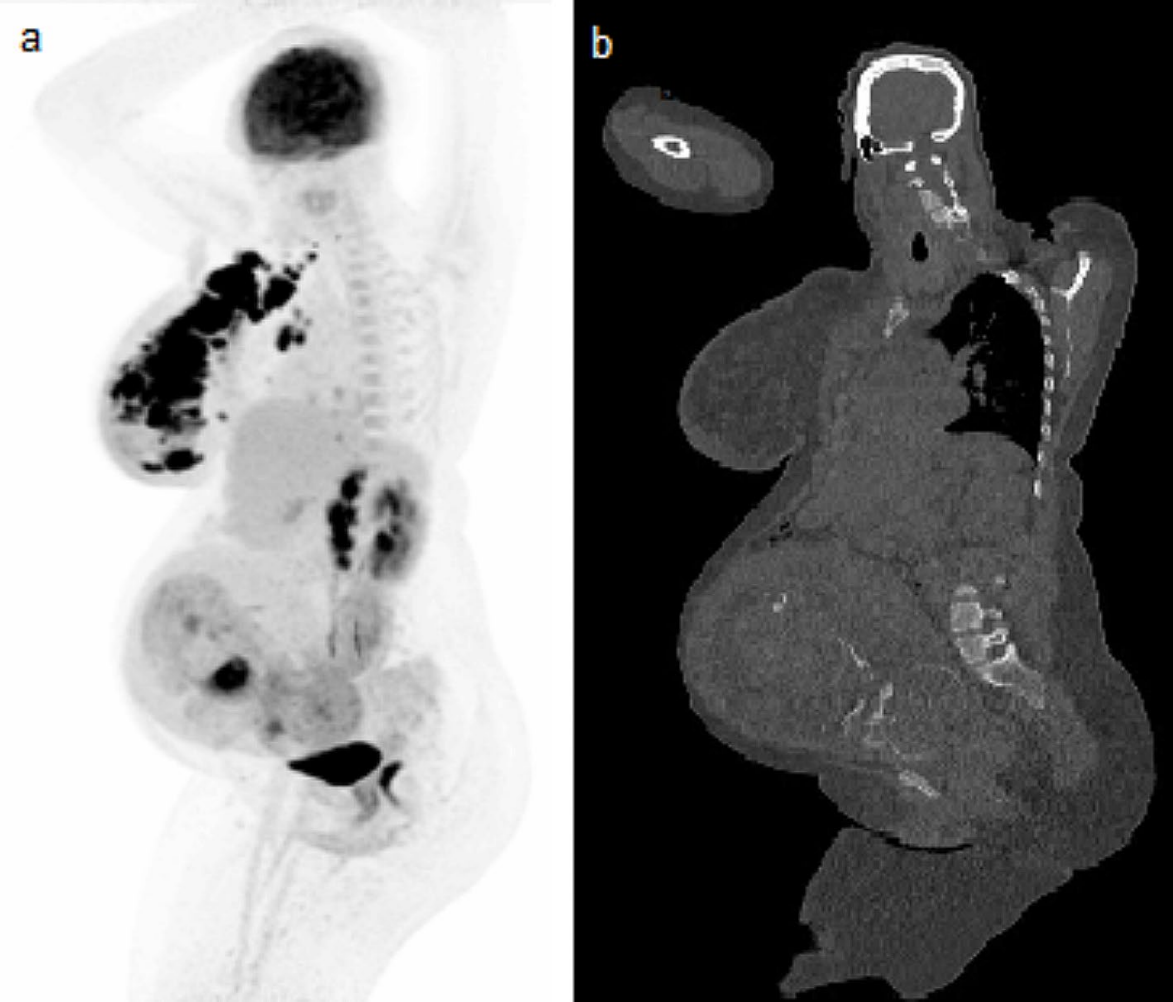
^18^F-FDG PET/CT image including both the mother and the foetus with **(a)** a Maximum Intensity Projection (MIP) ^18^F-FDG PET image and **(b)** a slice of the ultra-low-dose CT scan

Two pregnant patients in the second and third trimester with an indication for a clinical ^18^F-FDG PET/CT scan for staging of the disease were retrospectively included. Data was collected at the Amsterdam UMC, location VUmc from ongoing clinical investigations and the use of anonymized clinical data was waived by the VU Medical Centre ethics review board. Both scans were collected on the Siemens Vision Quadra PET/CT system (Siemens Healthineers, Knoxville, TN, USA) (LAFOV PET/CT system). The scans were acquired using one static bed position for 20 min. In one patient, the foetus was not included in the field-of-view (FOV) to further reduce foetal radiation exposure. The scan covered the skull vertex to the lower part of the kidneys with the head centred in the middle of the FOV. In the other patient, the foetus was included in the FOV and covered the skull vertex to mid-thigh (Fig. 1a, b).

### ^18^F-FDG PET

The patients received an intravenous bolus injection of 0.3 MBq/kg radioactive ^18^F-FDG and were fasting for 4–6 h before the scan. The scans were reconstructed with the European Association of Nuclear Medicine Research Ltd 2 (EARL-2) compliant reconstruction protocol [5, 7, 9]. Foetal radiation dose from radioactive ^18^F-FDG administration was estimated using dose conversion factors (mGy/MBq) from three published papers including second and third-trimester pregnancies [1, 3, 6]. All voiding models were included since the voiding pattern was unknown. Dose conversion factors from first-trimester pregnancies were excluded because foetuses in earlier stages absorb more radiation than foetuses in later stages of the pregnancy [3, 6, 8].

### Ultra-low-dose CT

An ultra-low-dose CT (LDCT) scan was applied to correct the PET scan for attenuation. A kilovoltage peak of 100 kVp, tube current of 20 mAs, 0.5 s rotation time and a pitch factor of 1.45 was applied for both CT scans. Radiation exposure to the foetus from the LDCT scans was based on the computed tomography dose index (CTDI) measured in milligray (mGy) and estimated using CT-Expo (version 2.7).

## Results

The dose conversion factors from the PET in the three studies ranged from 0.004 mGy/MBq to 0.014 mGy/MBq with a mean foetal radiation exposure of 0.008 ± 0.003 mGy/MBq. The dose conversion factors are summarized in Table 1.

**Table 1:** Dose conversion factors from radioactive ^18^F-FDG

Publication	Second trimester (mGy/MBq)	Third trimester (mGy/MBq)
Stabin et al., mean	0.014	0.007
Takalkar et al., mean (range)		
2 h - voids	0.008 (0.007–0.010)	0.009 (0.007–0.011)
Irregular voids	0.006 (0.006–0.007)	0.008 (0.006–0.011)
	**Late pregnancy (mGy/MBq)**	
Fregonara et al., mean (range)		
1 h – voids	0.007 (0.006–0.008)	
3.5 h – voids	0.005 (0.004–0.006)	

*Abbreviations*: MBq; mega Becquerel, mGy; milligray

The first patient received an ^18^F-FDG PET/CT scan for staging of a haematological malignancy. The patient received 27.58 MBq radioactive ^18^F-FDG, resulting in a foetal radiation dose from the PET ranging between 0.11 and 0.39 mGy, with a mean foetal radiation dose of 0.21 ± 0.07 mGy. The foetus was not included in the FOV, resulting in a foetal radiation dose from the LDCT scan of < 0.10 mGy due to scatter radiation. The total radiation dose on the foetus ranged between 0.11 and 0.49 mGy. The second patient received an ^18^F-FDG PET/CT scan for staging of a solid malignancy and received an intravenous bolus injection of 31.08 MBq radioactive ^18^F-FDG. The foetal radiation dose from the PET ranged between 0.13 and 0.44 mGy, with a mean foetal radiation dose of 0.24 ± 0.08 mGy. The foetus was included in the FOV, resulting in an estimated foetal radiation dose from the LDCT of 0.90 mGy. Therefore, the total foetal radiation dose ranged between 1.03 and 1.34 mGy. Foetal radiation doses of both patients and patient characteristics are summarized in Table 2.

**Table 2:** Patient characteristics and foetal PET/CT radiation dose estimates

Characteristics	Patient 1	Patient 2
Weight (kg)	77.0	88.5
Length (cm)	165	163
Body Mass Index	28.28	33.31
Injected activity (MBq)	27.58	31.08
Foetal dose PET (mGy), mean (range)	0.21 (0.11–0.39)	0.24 (0.13–0.44)
Foetal dose CT (mGy)	< 0.10	0.90
Total foetal dose (mGy), range	0.11–0.49	1.03–1.34

*Abbreviations*: MBq; mega Becquerel, mGy; milligray

## Discussion

This study aimed to report the foetal radiation dose when pregnant patients received an ^18^F-FDG PET/CT scan on a LAFOV PET/CT system with reduced injected activity.

We managed to scan patients with only 10% of the normal injected activity when conducting a PET/CT scan on a LAFOV PET/CT system while maintaining acceptable PET image quality. According to the physicians, the quality of the PET images was more than sufficient for disease staging in both cases. The quality of the LDCT scans was reliable for attenuation correction (AC) and provided sufficient quality for anatomical localisation of tracer uptake. The ultra-low administrated radioactivity led to a maximum foetal radiation exposure from the PET of 0.44 mGy. In our study, the estimated foetal radiation dose from a PET scan was noticeably lower compared to previous studies using SAFOV PET/CT systems [1, 6]. They observed a foetal radiation dose from the PET ranging between 1.01 and 2.68 mGy (mean 1.82 ± 0.55). Furthermore, we reduced the foetal dose from the LDCT scan for AC to 0.90 mGy when the foetus was included in the FOV. This is markedly lower compared with the study of Işıkçı et al. [4] that observed radiation exposure from CT scans on the foetus ranging between 8.5 and 16.0 mGy. Moreover, advancements such as the use of tin filters during LDCT acquisition could dramatically reduce radiation exposure for AC by as much as 90%, as recently demonstrated by Mostafapour et al. [10]. Applying the tin filter to our LDCT scans will reduce the foetal radiation exposure by eight to ten times, resulting in a foetal radiation exposure ranging between 0.09 and 0.11 mGy when the foetus is included in the FOV. The radiation dose to the foetus from an ^18^F-FDG PET/CT scan might then be considered imperceptible given that a foetal radiation dose below 1.0 mGy is generally considered safe and negligible according to existing literature [11]. Based on these findings, we can conclude that the ultra-high sensitivity of LAFOV PET/CT systems reduced the radiation dose to the foetus significantly and, in the future, the radiation exposure from an ^18^F-FDG PET/CT might be negligible for the foetus.

## Data Availability

The datasets generated during and/or analysed during the current study are available from the corresponding author upon reasonable request.
